# The Invadopodia Scaffold Protein Tks5 Is Required for the Growth of Human Breast Cancer Cells *In Vitro* and *In Vivo*

**DOI:** 10.1371/journal.pone.0121003

**Published:** 2015-03-31

**Authors:** Barbara Blouw, Manishha Patel, Shinji Iizuka, Christopher Abdullah, Weon Kyoo You, Xiayu Huang, Jian-Liang Li, Begoña Diaz, William B. Stallcup, Sara A. Courtneidge

**Affiliations:** Tumor Microenvironment and Metastasis Program, NCI Cancer Center, Sanford-Burnham Medical Research Institute, La Jolla, California, United States of America; University of South Alabama, UNITED STATES

## Abstract

The ability of cancer cells to invade underlies metastatic progression. One mechanism by which cancer cells can become invasive is through the formation of structures called invadopodia, which are dynamic, actin-rich membrane protrusions that are sites of focal extracellular matrix degradation. While there is a growing consensus that invadopodia are instrumental in tumor metastasis, less is known about whether they are involved in tumor growth, particularly *in vivo*. The adaptor protein Tks5 is an obligate component of invadopodia, and is linked molecularly to both actin-remodeling proteins and pericellular proteases. Tks5 appears to localize exclusively to invadopodia in cancer cells, and *in vitro* studies have demonstrated its critical requirement for the invasive nature of these cells, making it an ideal surrogate to investigate the role of invadopodia *in vivo*. In this study, we examined how Tks5 contributes to human breast cancer progression. We used immunohistochemistry and RNA sequencing data to evaluate Tks5 expression in clinical samples, and we characterized the role of Tks5 in breast cancer progression using RNA interference and orthotopic implantation in SCID-Beige mice. We found that Tks5 is expressed to high levels in approximately 50% of primary invasive breast cancers. Furthermore, high expression was correlated with poor outcome, particularly in those patients with late relapse of stage I/II disease. Knockdown of Tks5 expression in breast cancer cells resulted in decreased growth, both in 3D *in vitro* cultures and *in vivo*. Moreover, our data also suggest that Tks5 is important for the integrity and permeability of the tumor vasculature. Together, this work establishes an important role for Tks5 in tumor growth *in vivo*, and suggests that invadopodia may play broad roles in tumor progression.

## Introduction

Improvements in the early detection and the treatment of breast cancer have greatly reduced the mortality of the disease. However, the ability of tumor cells to infiltrate their surrounding microenvironment and wreak havoc on an otherwise uncompromised biological system underlies tumor metastasis, and remains the major cause of death in breast cancer patients. Our goal is to identify the molecular mediators of invasion in breast cancer cells that may warrant efficient and targeted drug design in the future.

Tumor cells are obliged to penetrate, remodel and degrade the extracellular matrix (ECM) in order to invade and metastasize [1]. One recognized mechanism for ECM degradation is the formation of dynamic, actin-rich structures called invadopodia, which in tissue culture form on the ventral surface of cells in contact with ECM and act as focal sites of its degradation [2–5]. Invadopodia were first noted in Src-transformed fibroblasts [6,7], and subsequently have been well described in many invasive human cancer cells, particularly those derived from breast cancer, melanoma and glioblastoma. A host of proteins are localized to invadopodia, including proteases, actin regulatory proteins and signaling molecules. Few, if any, of these molecules are selectively localized to invadopodia, however. This makes it challenging to separate the functions of invadopodia from those of other actin-based structures and protrusions. To circumvent this, many studies define invadopodia by the simultaneous presence of ECM degradative capacity, and key actin regulators such as cofilin or cortactin. However, it is not currently feasible to use these criteria to study the functions of invadopodia *in vivo*.

Some years ago, we identified a novel Src substrate and scaffold protein called Tks5, and showed that it both localized to, and was required for the formation of, invadopodia [8–10]. A number of Tks5 binding partners have been identified which can link Tks5 to both actin-remodeling proteins and pericellular proteases; these include ADAM-family metalloproteases [9], and the adaptor proteins Grb2, nWASP and Nck2 [11,12]. Importantly, Tks5 appears to be localized exclusively to invadopodia in cancer cells, and our *in vitro* studies demonstrated its critical requirement for the invasiveness of breast cancer cell lines [8]. This prompted us to investigate the requirement for Tks5 for tumor growth and metastasis *in vivo*, initially using the experimental system of Src-transformed mouse fibroblasts. We found that knockdown of Tks5 using RNA interference impaired the tumorigenicity of the cells when introduced by either subcutaneous or intravenous routes, perhaps by impairing tumor angiogenesis [13]. These studies suggest that invadopodia-mediated invasive behavior may be pivotal not just for metastatic progression (for example intra- and extravasation), but may also impact the growth of the tumor.

Recently, three important studies have demonstrated the clinical prognostic relevance of Tks5 expression in glial-derived brain tumors [14], lung adenocarcinomas [15] and prostate cancer [16], further justifying more detailed *in vitro* and *in vivo* analysis of Tks5 function. Here we undertook an evaluation of Tks5 expression and function in breast cancer.

## Methods

### Ethics

This study was approved by the Sanford-Burnham Medical Research Institute Animal Care and Use Committee (AUF protocols 09–146 and 12–096), and performed in accordance with the Institute of Laboratory Animal Research (NIH, Bethesda, MD) Guide for the care and Use of Laboratory Animals. The ARRIVE Guidelines Checklist-NC3Rs for Animal Research is provided in S1 ARRIVE Checklist. The Sanford-Burnham Medical Research Institute Institutional Review Board did not require approval and waived the need for consent for the breast cancer progression tissue microarrays from the Cancer Diagnosis Program (CDP) of the National Cancer Institute following approval from the National Disease Research Interchange (NDRI). NDRI gains consent for the de-identified samples that they provide.

### Antibodies for immunoblotting and immunohistochemistry

Antibody dilution and catalog numbers for the antibodies used for immunoblotting are shown in parentheses. The antibody for Tks5 (1:2000; anti-Tks5 1737) was generated by the Courtneidge laboratory. The anti-Tubulin γ (1:5000; T6557) antibody was from Sigma. Antibodies used in immunofluorescence include: anti-Tks5 antibody (1:10; anti-Tks5 1737) used with a secondary antibody [1:200; Alexafluor-594-conjugated anti-mouse (Chemicon)]. Sections were counter-stained with Hoechst to identify the nuclei (Sigma, St. Louis, MO) (1:10,000). To determine vessel morphology and density, frozen sections were stained with mouse anti-CD31 (BD Biosciences) (1:100) in conjunction with a secondary antibody [1:100; Alexafluor-594-conjugated anti-mouse (Chemicon)]. Anti-VEGF was purchased from R&D Systems (1:10) and the secondary was Alexafluor-594-conjugated anti-mouse (Chemicon). Ki-67 was obtained from Dako (1:100) and the secondary was Alexafluor-594-conjugated anti-mouse (Chemicon). TUNEL staining was carried out according to the manufacturer’s instructions of ApopTag Fluorescein Direct In Situ Apoptosis Detection Kit (Chemicon). The hypoxia probe injection and staining of frozen sections was carried out as described [17]. For the immunohistochemistry of breast cancer specimens, the Tks5 antibody from ProteinTech was used according to manufacturer’s specification.

### Plasmids

shRNAs pLKO.1 lentiviral plasmids used for scrambled and human Tks5 knockdown were purchased from Sigma. The clones used were TRCN0000136014 (targeting the 3’ UTR, and referred to as clone D6) and TRCN0000136512 (targeting the coding region, and referred to as clone D7). Human Tks5-GFP was generated via subcloning into the BglIII/BsrGI sites of the SIN18-PGK-GFP vector [made at the Sanford-Burnham Medical Research Institute (SBMRI)]. The doxycycline-inducible shRNA lentivirus was generated previously by D. Wiederschain (Novartis Developmental and Molecular Pathways, Cambridge, MA) and generously donated to our lab [18]. The D6 clone that created the most efficient Tks5 knockdown was cloned into the AgeI/EcoRI sites of the doxycycline-inducible lentivirus according to the instructions provided by D. Wiederschain.

### Immunohistochemistry of tumor specimens

The Tks5 antibody (ProteinTech) was first evaluated for specificity and optimal titer by using it to stain paraffin embedded 293 cells transfected with empty vector, or vectors for the adaptor proteins Tks4 and Tks5. The antibody was then used to stain a breast cancer progression tissue microarray obtained from the Cancer Diagnosis Program (CDP) of the National Cancer Institute following approval from the National Disease Research Interchange (NDRI) (http://ndriresource.org/). Antigen retrieval was performed according to the manufacturer’s guidelines using Antigen Retrieval Buffer (DAKO S1699). After incubation with the Tks5 antibody, the sections were incubated with secondary antibody using the manufacturer’s guidelines of the MACH2 Rabbit AP-Polymer (Biocare Medical) and visualized with either Vulcan Fast Red Chromogen (Biocare Medical) (pink) or DAB (brown). Sections were counterstained with hematoxylin. A scoring system was developed of 0, no staining; 1+, low staining or staining in <25% of tumor cells; 2+, moderate staining; and 3+, high staining. Slides were independently evaluated by a trained pathologist (S.I.).

### Analysis of RNA sequencing data

RNA sequencing data from the breast cancer (BRCA) dataset of The Cancer Genome Atlas (TCGA) sequencing project (https://tcga-data.nci.nih.gov/tcga/), produced at the University of North Carolina, and the accompanying clinical data, were used for the analysis [19]. RNAseq reads for each BRCA sample aligned to Tks5 were obtained and used to calculate the depth of coverage for each exon in the Tks5 gene. Tks5 total expression was measured by summing the depth of coverage over exons 1–14, and Tks5α (long form) expression was measured by summing the depth of coverage over exons 1–5. Median value of Tks5 total expression or Tks5α (long form) expression of all BRCA samples was used as cutoff to divide all samples into High (> median, n = 425) and Low (<median, n = 425) groups.

### Cell culture and infections

The luciferase-expressing human breast cancer cell line MDA-MB-231-Luc was obtained from Xenogen and was routinely cultured in Dulbecco’s minimal essential medium (DMEM) supplemented with 10% Fetal Bovine Serum (FBS) and a cocktail of penicillin and streptomycin (P/S) (Gibco). MDA-MB-231-Luc cells stably expressing scrambled or Tks5 knockdown were generated by infection using scrambled (Scr), D6 or D7 shRNA viruses (generated by the core facility at SBMRI). For rescue experiments, shRNA D6-expressing MDA-MB-231-Luc cells were stably infected with Tks5-GFP. Following infection, cell populations were selected and maintained in DMEM/10%FBS/P/S/5μg/ml puromycin.

### 3D proliferation and tumorsphere assays

For proliferation assays, 18000–25000 MDA-MB-231-Luc cells stably expressing Scr or D6 shRNA were incorporated into the non-solidified solution of native 3.8–4.1 mg/ml collagen I (BD Biosciences; Cat#354236), according to manufacturer’s instructions. Assays were allowed to progress for 7–10 days, with cell media replaced every 2–3 days. 3D tumorsphere growth assays were performed as described in [20]. Briefly, 3500 cells (per 8-well Labtek chamber) were plated on solidified Matrigel (without growth factors). Cells were overlaid with 2% Matrigel (without growth factors) in 10%FBS/DMEM/P/S. Assays were allowed to progress for 7–10 days and cell media was replenished with fresh media every 4 days. Fixation and staining protocols were followed as in [21].

### Mice

All animal experiments were conducted in accordance with the NIH Guide for the Care and Use of Laboratory Animals and were approved by the SBMRI Animal Care and Use Policy. In all experiments, SCID-Beige mice were obtained from Harlan and housed in a mouse Specific Pathogen Free (SPF) facility. SCID-Beige mice were acquired from the Jackson Laboratory. After quarantine, SCID-Beige mice were maintained under standard pathogen-free conditions in a constant temperature and humidity environment with a 12:12 light:dark cycle and *ad libitum* access to food and water at the Sanford-Burnham Medical Research Institute Animal Facility, La Jolla, California. They were continuously monitored during daytime from Monday to Friday, and twice daily during daytime on Saturdays, Sundays, and holidays for signs of poor health. If mice showed signs of poor health, such as shakiness and low activity with decreased body temperature, they were humanely euthanized. For animals older than 7 days: CO_2_ inhalation until mice stop breathing, followed by cervical dislocation. These methods have been approved by the Sanford-Burnham Medical Research Institute Animal Care and Use Committee following the American Veterinary Medical Association guidelines for the Euthanasia of Animals (https://www.avma.org/KB/Policies/Documents/euthanasia.pdf, sections S2.2.2.1, S2.2.2.3 and S2.2.4.2.2, and http://grants.nih.gov/grants/olaw/Euthanasia2007.pdf).

### Orthotopic mammary fat pad injections and lung metastasis analyses

Mammary fat pad injections in the number 4 mammary gland were carried out according to Dunphy and colleagues without the clearing of the fat pad [22]. Briefly, cells were harvested by trypsinization and resuspended in PBS (Invitrogen). For each cell line, female SCID-Beige mice were injected in a non-cleared mammary fat pad with 1x10^6^ to 2x10^6^ cells per animal in a volume of 25μl [1:1 ratio with Matrigel (BD Biosciences)], and tumors were allowed to form for ~30 days. Tumor onset was determined by physical palpation. Tumor growth was measured every 2–3 days using calipers; both the longest (L) and shortest (S) measurements were recorded. Using these values, tumor volumes were calculated as follows: (L×S2) × 0.5, and expressed as mean volume ± SEM. Mice were sacrificed when the tumors reached a diameter of 2cm, according to the Animal Care and Use Policy of the SBMRI. These experiments were repeated at least 3 times, using 5–10 mice per tumor group. For the lung metastasis analyses, mice were observed for signs of tumor burden such as loss of weight and a hunched back. After ~30 days, mice were sacrificed and the lungs were harvested and fixed in 4% paraformaldehyde. Metastatic lesions per lung were analyzed under a dissection microscope (Zeiss). This experiment was repeated 3 times using 5–10 mice per group. One-way ANOVA or student t-tests were performed on the tumor volume at the endpoint of the experiment to determine statistical significance.

### Doxycycline treatment

Doxycycline was administered to cells either *in vivo* or *in vitro* in cell culture. For the *in vivo* model, after orthotopic implantation of cells, induction of Tks5 knockdown was started (at various time points) by adding doxycycline (2mg/ml) plus 2% sucrose to the drinking water, whereas control animals received 2% sucrose alone. For *in vitro* cell treatment, cells were exposed to doxycycline (500ng/ml) for 10 days to induce Tks5 knockdown in these cells. The mice injected with these cells and control cells were then continuously exposed to doxycycline via the drinking water as mentioned above. Mice were inspected daily for body weight loss, general condition, and tumor formation via palpation. All animals tolerated the procedure well. Primary tumors were analyzed and processed as indicated above (see section Orthotopic mammary fat pad injections and lung metastasis analyses).

### Determination of vascular leakage

FITC-Dextran (250kDa; Sigma) was injected intravenously (0.1ml of a 50mg/ml solution) via the tail veins of Avertin anesthetized mice bearing scrambled control and Tks5 knockdown tumors [17]. After 10 minutes to allow for circulation, mice were cervically dislocated while still under anesthesia. Tumors were removed without perfusion and fixed overnight in 1% paraformaldehyde. After cryoprotection in 20% sucrose, material was frozen in OCT compound. Cryosections were prepared and immunostained for CD31. The extent of FITC-Dextran leakage from blood vessels was quantified on the basis of green fluorescence located external to CD31-positive vascular structures. Using Image-Pro Plus 4.5 software, the borders of CD31-labeled blood vessels were identified so that extravascular FITC-dextran could be quantified by counting green pixels external to red blood vessels. Vascular leakage was defined as extravascular FITC-Dextran/total FITC-Dextran. At least 5 microscopic fields were examined in 4–5 tumors from each genotype by confocal microscopy.

### Tumor Hypoxia

Mice bearing control and Tks5 knockdown tumors were injected intravenously under Avertin anesthesia with 60 mg/kg pimonidazole hydrochloride [17]. After a 60-minute incubation period, mice were perfused with 1% paraformaldehyde, and tumors were processed for immunocytochemistry using FITC-conjugated mouse anti-pimonidazole antibody (1:100, Hydroxyprobe, Inc). Levels of hypoxia were determined by quantifying green pixels per tumor area.

### Histology

Tumor and lung tissue was flash-frozen and processed as previously described in [13]. For immunohistochemistry and H&E analysis, tissues were sectioned and stained. Lung sections were collected to determine metastases incidence and quantification, and photographs were taken with a 4× objective. To determine cell death, apoptotic cells of both frozen and paraffin sections were detected using the ApopTaq Fluorescein Direct In Situ Apoptosis Detection Kit purchased from Chemicon and used according to the manufacturer's instructions. Nuclei were identified with Hoechst 33258. Photographs of Tks5 immunofluorescence and apoptosis were taken at a magnification of 200x on an Axioplan 2 fluorescence microscope and were analyzed with Axiovision 3.0 software (Zeiss). Images of the PCNA and CD31 staining were taken at magnifications of 100x and 200x, respectively. The photographs of the H&E images were taken at a magnification of 40x. All images were generated with an inverted TE300 Nikon Fluorescence Microscope with a CCD Spot RT Camera using Spot RT Acquisition and Processing Software (Diagnostic Instruments Inc, Sterling Heights, MI).

### Quantification of histological assays

For the quantification of the proliferation rate, apoptotic rate, cellular density, vessel density and dilation, ImagePro software was used (MediaCybernetics, Bethesda, MD) and analyses were performed as in reference [13]. For each analysis, tumors/lungs obtained from at least 2 to 3 different mice per group were used, and at least 6–7 photographs were taken per tumor/lung.

### Statistics

RNA sequencing and clinical data: Fisher’s exact tests were performed to examine the association between Tks5α expression levels and patients with different disease stages, tumor type, grade, etc. Overall survival was analyzed by Kaplan-Meier curves and log-rank test for all BRCA patients and those patients with more than 5 years survival. Multivariate analysis by the Cox proportional hazard model (adjusted by age, tumor subtype, and pathological stage) was performed to assess survival results. P-values (two-tailed) of <0.05 were considered statistically significant.

In vitro experiments: All numerical data are shown as mean ± SD. Error bars on all graphs represent the standard deviation between measurements.

*In vivo* experiments: Statistical significance was determined by student’s t test, or two-way repeated ANOVA with Bonferroni post-hoc test, as appropriate.

## Results

### Tks5 expression in breast cancer specimens

**Fig 1:**
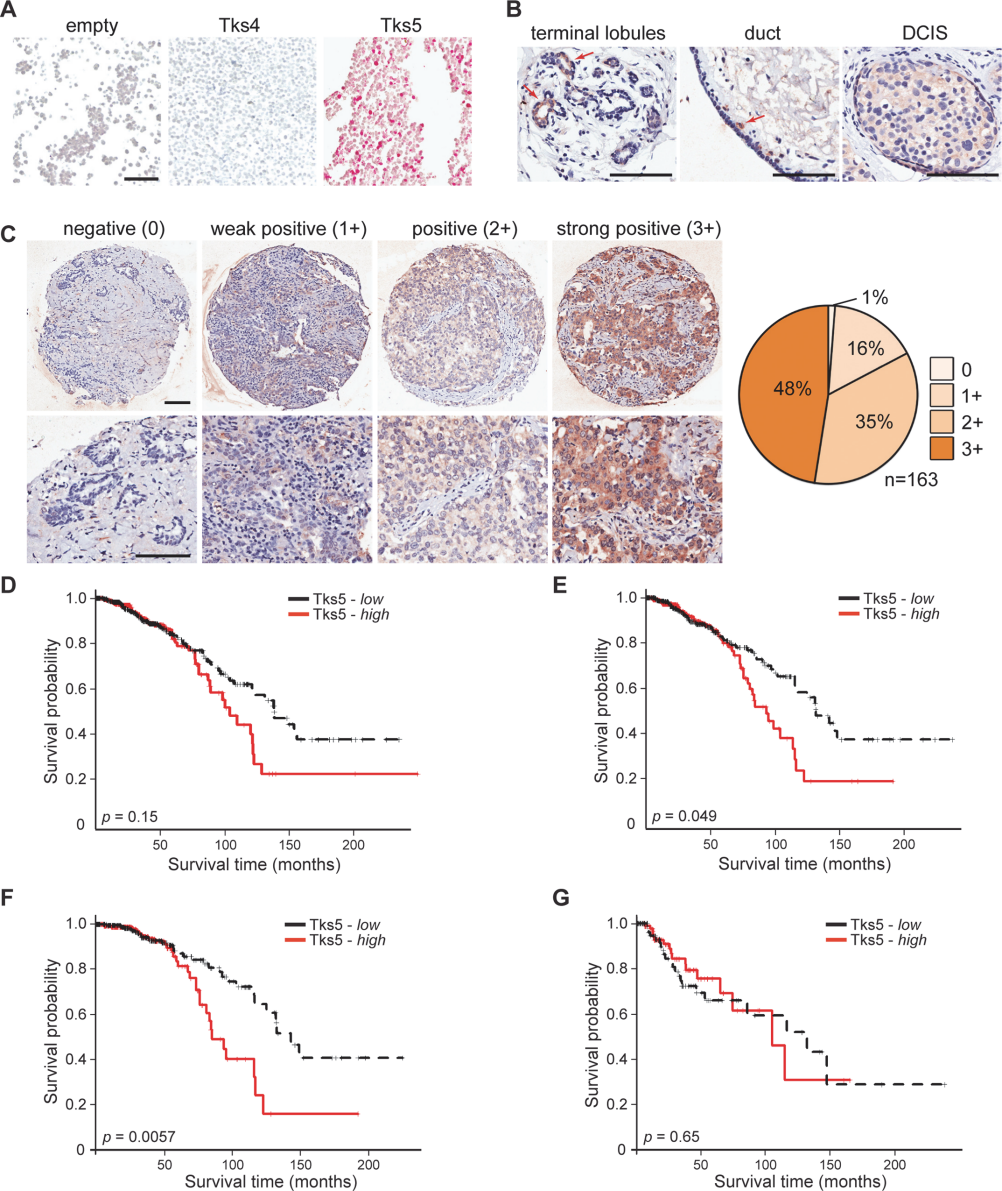
Tks5 expression in breast tissue and breast cancer. A) 293 cells were transfected with empty vector, or vectors expressing Tks4 or Tks5. Cells were pelleted, fixed, embedded in paraffin and processed for immunohistochemistry with an anti-Tks5 antibody. B) Representative images of normal breast lobules and duct, and ductal carcinoma in situ, stained with anti-Tks5 antibodies. Red arrows indicate areas with intense Tks5 staining. Scale bar represents 100μm. C) Representative images of primary invasive breast cancer specimens, at low and high magnification, to illustrate the range of Tks5 expression observed. Scale bars represent 100μm. Quantification of 163 specimens is shown on the right. D) Kaplan-Meier survival curves for patients with high (red) and low (black) Tks5α mRNA levels. E) Kaplan-Meier survival curves for stage I/II patients with high (red) and low (black) Tks5α mRNA levels. F) Kaplan-Meier survival curves for stage III/IV patients with high (red) and low (black) Tks5α mRNA levels.

To explore the clinical relevance of Tks5 expression in breast cancer, we first conducted immunohistochemical (IHC) analysis on a cohort of human breast cancer tissues provided by the Cancer Diagnosis Program of the National Cancer Institute. We used a commercial rabbit polyclonal antibody which we first validated for specificity for Tks5 using transfected cell populations (Fig 1A). The same batch of antibody, and the same staining protocol, was used on the breast cancer tissue microarrays (TMA), and a scoring system was developed. These TMAs also contained a few (<20) specimens of normal breast tissue and ductal carcinoma in situ (DCIS). Normal breast tissue was largely negative or 1+ for Tks5 expression, although we did detect occasional terminal lobules, and isolated ductal cells, which were strongly positive (Fig 1B). The DCIS specimens were largely 1+, although larger sample sizes would be needed to judge the reliability of this finding. Of the 163 evaluable primary invasive breast carcinoma specimens, only 1% showed no staining, 16% were 1+, 35% were 2+, and 48% were 3+ (Fig 1C). These findings support further study of Tks5 in breast cancer. However, batch-to-batch variability of the commercial antibody made us reluctant to use it for IHC of larger datasets to explore any association with tumor type and/or outcome. We therefore decided to use RNA sequence (RNAseq) analysis for our next experiments.

Recent studies have shown that there are multiple forms of Tks5, generated by alternative promoters ([15,23]). The long form (Tks5-long or Tks5α) refers to the full length protein with an amino-terminal PX domain followed by 5 SH3 domains. There are also short forms of Tks5 (Tks5-short, Tks5β and others) which are initiated at different, internal, promoter(s) and lack the PX domain. Only the PX domain-containing form of Tks5 can contribute to invadopodia formation, and in tissue culture, human cancer cell lines express only or predominantly this long, Tks5α, form [8,11]. However, many primary human tumor samples do express short form mRNAs. Moreover, it was recently shown in lung cancer that a high ratio of long to short forms of Tks5 is associated with a worse prognosis [15]. We first used the methodology described in [15] to examine the breast cancer RNAseq dataset from the University of North Carolina [19]. We found no statistically significant association between either total Tks5 expression, or a high long/short ratio, and tumor type, grade or outcome (data not shown). We therefore focused our analysis on the long (Tks5α) form, by analyzing the expression of the first 5 exons, which encode the PX domain. Median Tks5α expression values were used as the cutoff to divide all patients into High (>median, n = 425) and Low (<median, n = 425) groups. First, patients were divided into stage I/II and stage III/IV groups, with Fisher’s exact test used to determine any association. No significant association was found between Tks5α expression and disease stage (p = 0.56). In terms of breast cancer type, 56% of ER-positive, 51% of Her2-positive, and 38% of triple negative breast cancers were in the High group for Tks5α expression (p = 0.0013). Next, Kaplan-Meier curves and Log rank tests were used to evaluate any overall survival differences between High and Low Tks5α expressors. There was a significant difference in overall survival between the two patient groups, with a median survival time of 93.3 months for the High group and 132.0 months for the Low group (p = 0.049) (Fig 1D). It is noteworthy that the two survival curves overlap for the first 60 months. Beyond this time, the negative association between high Tks5α expression and survival becomes highly statistically significant (n = 142, p = 0.00078). Interestingly, for patients with stage I/II disease, median survival time for the High group was 84.5 months, and for the Low group 142.4 months (n = 626, p = 0.0057) (Fig 1E), whereas for patients with stage III/IV disease, differences in median survival for the High group (104 months) and the Low group (131 months) were not statistically significant (n = 205, p = 0.65) (Fig 1F). Multivariate analyses were also conducted. High Tks5α expression is still significant (p = 0.012) after adjusting for age in stage I and II patients, but not if more variables are adjusted. However, high Tks5α is a significant prognostic indicator (p = 0.017) after adjusting for age, BRCA subtype, and pathological stage in patients surviving more than 5 years post diagnosis. In summary, these analyses suggest that overall survival of patients with pathological stage I and II breast cancer is significantly influenced by Tks5α expression level, particularly for those more than 5 years post diagnosis.

### Tks5 expression is required for tumor cell growth *in vitro* and in an orthotopic *in vivo* mouse model

**Fig 2:**
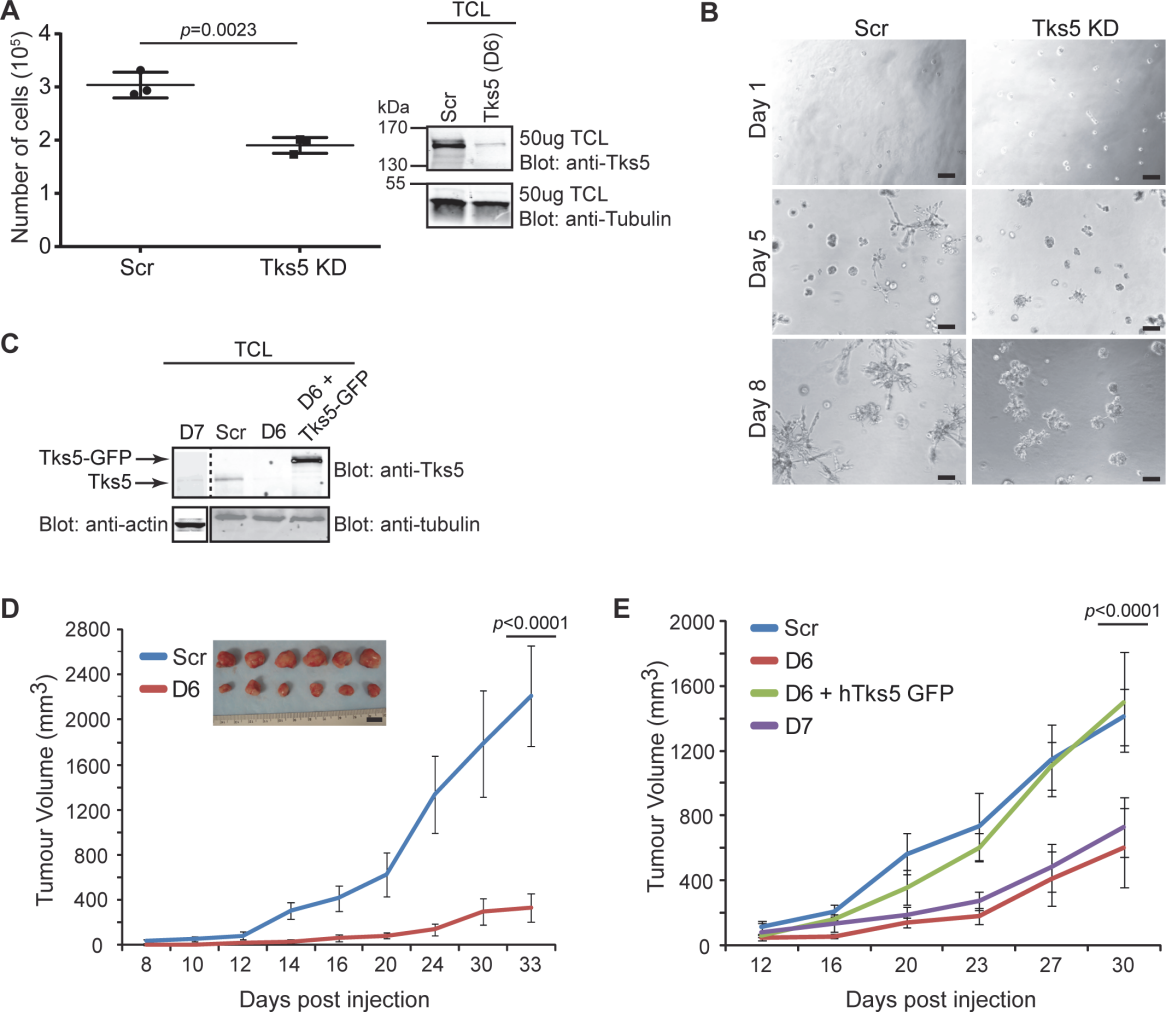
Reduced expression of Tks5 leads to decreased growth of tumor cells *in vitro* and *in vivo*. A) MDA-MB-231-Luc infected with either a scrambled (Scr) or Tks5 (D6) shRNA via lentivirus were subjected to growth assays in 3D conditions for 7–10 days. Immunoblot shows shRNA Tks5 knockdown (KD) compared to scrambled control in the cells used to evaluate growth. D6 = Tks5 knockdown clone. Experiments were performed in triplicate. B) Representative images of day 1, 5, and 8 MDA-MB-232-Luc [Scr and Tks5 KD (D6)] acini in 3D matrigel assays. Shown are low magnification phase-contrast images. Experiment was performed in triplicate. Scale bar: 100μm. C) Immunoblot: D6 and D7 represent different shRNAs tested for effective Tks5 knockdown in MDA-MB-231-Luc cells. These cell lines, as well as Scr and rescue (D6+hTks5-GFP) lines, were used for orthotopic injections in Fig 1D and 1E. D) Control (Scr) and Tks5 (D6) KD clones were inoculated in the mammary fat pad of SCID-Beige mice. Tumor volumes were measured every 2–3 days as described in Materials and Methods and tumors were allowed to grow to a final volume of approximately 2cm^3^. n = 20 mice per tumor type. Experiments were performed in triplicate (at least). Inset: Photographs of 6 tumors per group at the day of dissection (endpoint of experiment). Scale bar: 1cm. Tumor images are representative for all experiments performed. E) Control (Scr), Tks5 (D6 and D7) KD clones, and a Tks5 rescue clone (D6+ hTks5-GFP) were inoculated in the mammary fat pad of SCID-Beige mice. Tumor volumes were measured as in Fig 1D. n = 5 mice per tumor type. Experiments were performed in triplicate (at least). Data are expressed as mean ± SD. One-way ANOVA or a Student’s t test was used to calculate *p* values.

Our previous findings demonstrated that Tks5 is required for breast cancer cell invasion and ECM degradation *in vitro* [8]. Here, we wanted to determine whether the Tks5 had any effect on growth *in vitro*. We used lentiviral shRNA to knockdown Tks5 expression in a human breast cancer cell line MDA-MB-231 expressing luciferase (MDA-MB-231-Luc cells), and confirmed via immunoblot the extent of the knockdown (Fig 2A). Prior experiments revealed that Tks5 knockdown did not affect proliferation rates on tissue culture plastic when compared to controls [8]. Therefore we chose to conduct these experiments in three-dimensional (3D) matrices of native type I collagen. We observed a decrease in 3D cell growth for Tks5-KD cells as compared to scrambled knockdown cells (Fig 2A). We also evaluated these cells in the well-established 3D tumorsphere assays using matrigel. Normally in this assay, non-transformed breast epithelial cells (such as MCF10A) display low proliferation and remain stable in terms of acinar size and shape, with acini resembling hollowed-out spheroids. In contrast, tumor cells such as MDA-MB-231 exhibit high proliferation and form extensive stellate structures that often bridge multiple cell colonies [21,24]. We found that MDA-MB-231-Luc cells expressing scrambled shRNA developed the transformed morphology previously reported. Conversely, while Tks5-KD cells did not exhibit a full reversion to the normal acinus formation seen with MCF10A cells, these cells produced only small and poorly organized clusters with less growth and fewer protrusions (Fig 2B). Collectively, these data point towards a critical context-dependent effect of Tks5 on tumor cell growth *in vitro*.

We next wanted to study in more detail the effects of Tks5 on tumor growth and metastasis *in vivo*. Our previous research demonstrated that Tks5 is required for tumor growth of Src-transformed fibroblasts using both subcutaneous and tail-vein injection mouse allograft models [13]. Here we evaluated orthotopic injection of the MDA-MB-231-luc cell line, expressing scrambled control (Scr) or two distinct shRNAs specific for Tks5 (referred to as D6 and D7). Knockdown was confirmed by immunoblotting (Fig 2C), then cells were injected into the mammary fat pads of immunocompromised SCID-Beige female mice. We observed a large difference in tumor volume between mice implanted with Scr versus D6 cells (p<0.0001 by student’s t test), with Tks5 KD groups displaying approximately 5 to 7-fold decreases in tumor volume (Fig 2D). This decrease in tumor growth was also observed with the D7 Tks5 KD cells (p<0.0001 by two-way repeated ANOVA with Bonferroni post-hoc test) (Fig 2E), suggesting that the Tks5 effect on tumor growth is not due to off-target effects of the shRNA. To verify this, we also performed a rescue experiment in which MDA-MB-231-luc cells expressing the D6 Tks5 shRNA (which targets the 3’ UTR of the endogenous mRNA) were engineered to re-express a GFP tagged form of Tks5 (Tks5-GFP) (Fig 2C and 2E). We observed a rescue in tumor growth, with tumor volumes very similar to those from Scr control animals (p>0.05 by two-way repeated ANOVA with Bonferroni post-hoc test) (Fig 2E). Together, these experiments rule out off-target effects, and demonstrate that Tks5 is an essential player in *in vivo* tumor growth.

**Fig 3:**
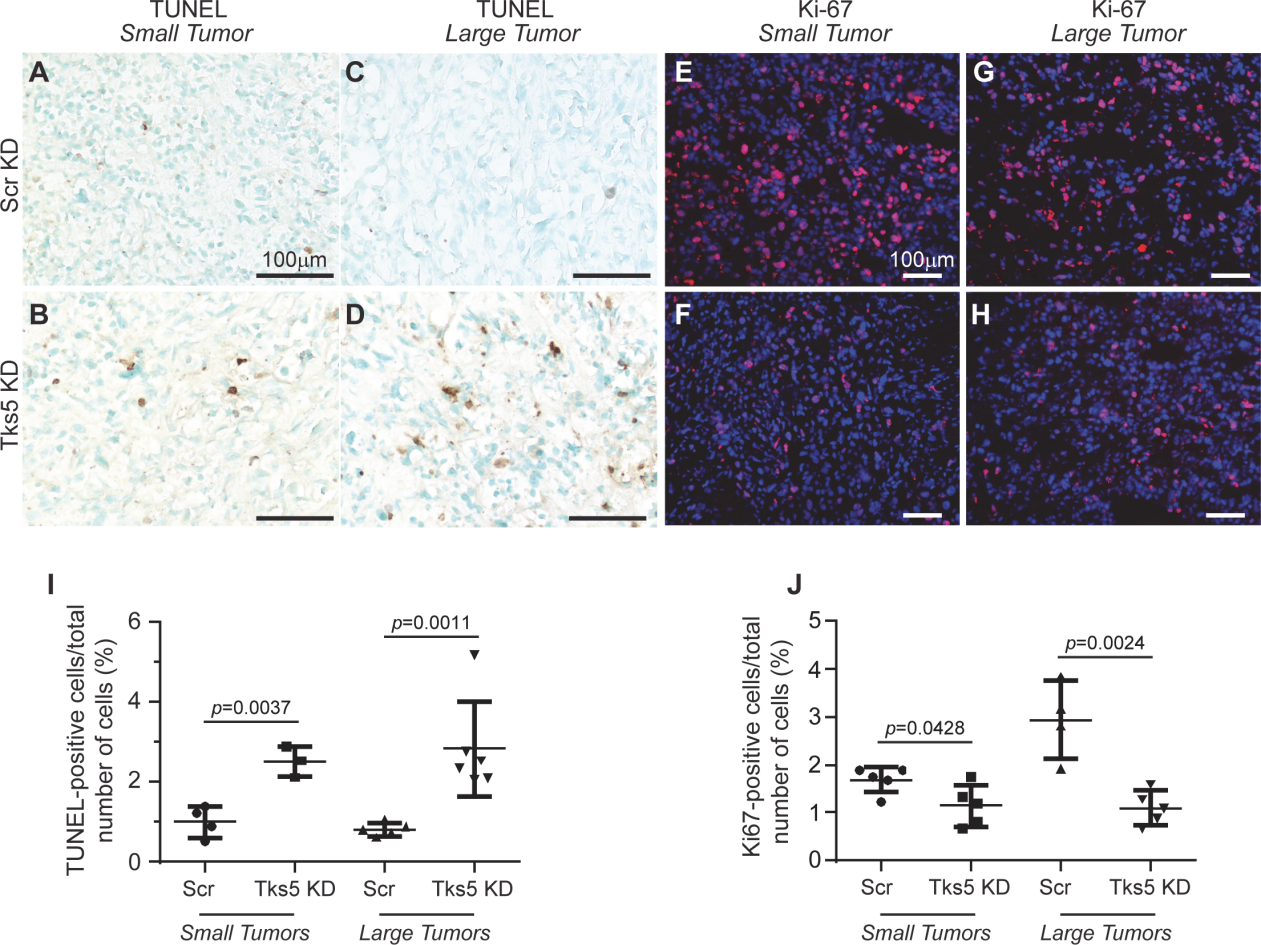
Tumor proliferation and apoptosis is affected by Tks5 knockdown. Tumors from Scr KD and Tks5 KD MDA-MB-231-Luc-orthotopic mouse models in Fig 2 were analyzed via immunohistochemistry and immunofluorescence, using size-matched tumors. A-D) TUNEL staining was used to visualize cell death in small (panels A-B) and large (panels C-D) tumors from Scr (panels A, C) and Tks5 (panels B, D) KD mice. Scale bar: 100μm. E-H) Ki-67 staining (nuclear protein marker) was used to visualize cell proliferation in small (panels E-F) and large (panels G-H) tumors from Scr (panels E, G) and Tks5 (panels F, H) KD mice. Scale bar: 50μm. Images are representative for all experiments performed. I-J) Quantification of positive immunohistochemical and immunofluorescence staining. Graphs show immune-positive cells for apoptosis (TUNEL) (panel I) and proliferative cells (Ki67) (panel J) at the day of dissection (endpoint of experiment). Data are expressed as mean ± SD. One-way ANOVA or a Student’s t test was used to calculate *p* values.

We next sought to explain the decrease in tumor growth *in vivo* in Tks5 KD tumors, determining first whether proliferation and/or apoptosis in the tumors was affected. To do this, we analyzed size-matched tumors with comparable tumor volumes at day 12 post injection. We noted that reduced expression of Tks5 in orthotopic tumors correlated with both an increased apoptotic rate (~2 to 3-fold change) and a decreased proliferation rate (~0.5 to 3-fold change) compared to scrambled controls, as shown by TUNEL and Ki-67 staining respectively (Fig 3). Similar analysis of larger-sized tumors showed analogous results (Fig 3). Together, our results suggest that Tks5 expression in tumor cells is integral for primary tumor proliferation and offers a defense against cell death.

### Reduced Tks5 expression correlates with decreased angiogenic properties in tumors

**Fig 4:**
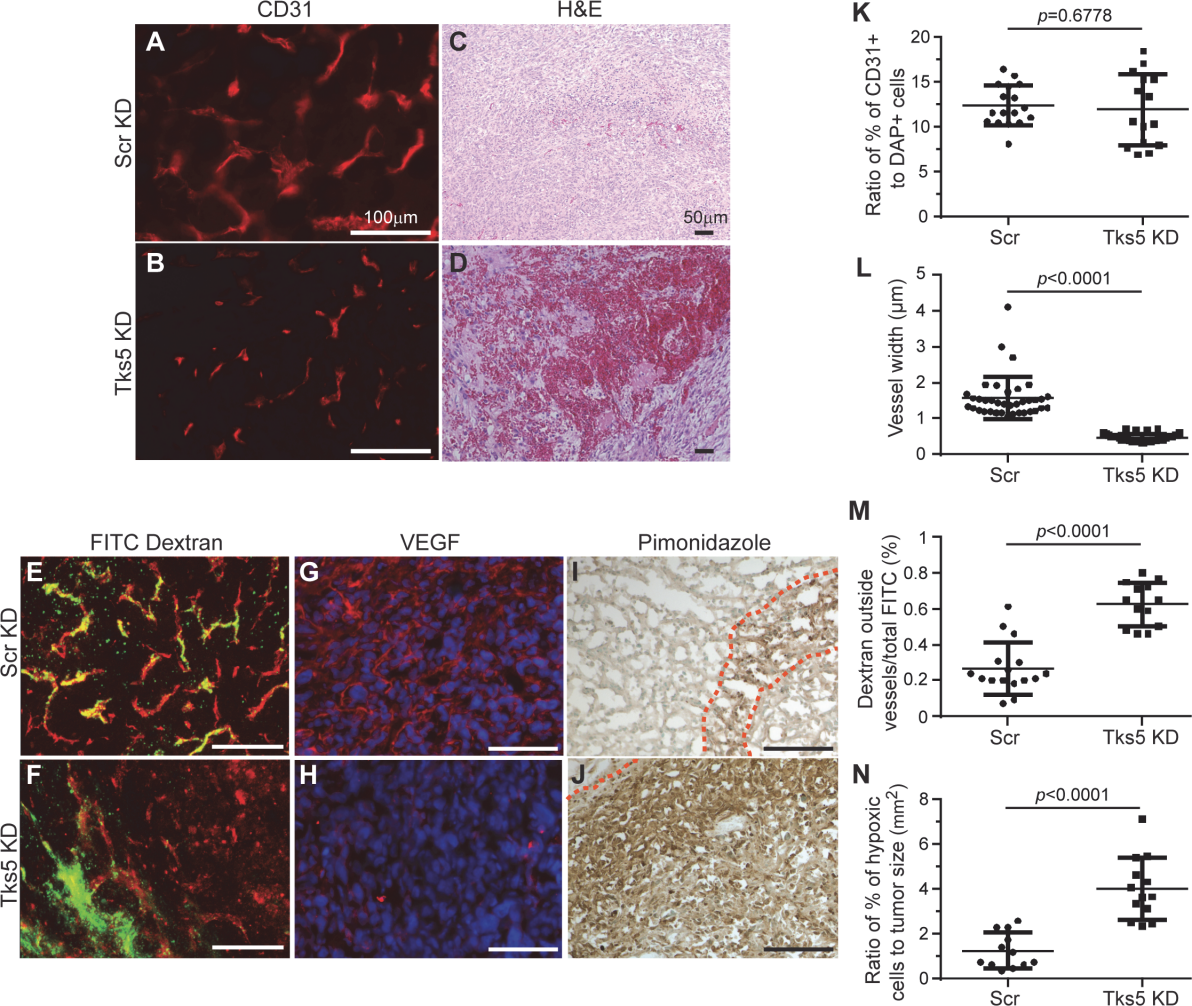
Reduction of Tks5 expression in tumor cells is associated with decreased angiogenesis. Tumors from Scr KD and Tks5 KD MDA-MB-231-Luc-orthotopic mouse models in Fig 2 were analyzed via immunohistochemistry and immunofluorescence in size-matched tumors. A-B) Vessel morphology and density was examined by staining tumor samples with CD31 (quantification panels K and L). C-D) Hematoxylin and eosin staining revealed altered vessel morphology and hemorrhaging in Tks5 KD tumors as compared to Scr KD tumors. Tumors were also analyzed for FITC-Dextran leakage (panels E-F, red = CD31; Green = FITC-dextran; quantification panel M), VEGF expression (red; panels G-H), and hypoxic areas (pimonidazole staining in panel I-J, quantification panel N). Red dashed lines delineate borders for areas of hypoxia. Scale bar: 100 μm, except panels C and D where scale bar: 50μm. Images are representative for all experiments performed. Data were expressed as mean ± SD. One-way ANOVA or a Student’s t test was used to calculate *p* values.

Cancer progression relies heavily on angiogenesis. This ability of tumors to promote new vasculature is essential for the transport of nutrients to the tumor mass, and is considered a rate-limiting step in tumor expansion [25]. Our previous study of Src-transformed fibroblasts suggested a role for Tks5 in eliciting tumor angiogenesis [13]. We therefore evaluated angiogenesis in the breast cancer model. Because the size of the tumors did not influence the proliferation or apoptotic rate, both small and large tumors were used for these analyses. We observed that Tks5 KD tumors exhibited narrower blood vessels compared to scrambled controls, but similar vessel density (Fig 4A, 4B, 4K and 4L). This altered vessel morphology in Tks5 KD tumors was associated with reduced vessel function as shown by an increase in tumor hemorrhaging (Fig 4C and 4D), as well as by an increase in FITC-Dextran leakage (Fig 4E, 4F and 4M). We also noted that Tks5 KD tumors exhibited reduced expression of VEGF in comparison to scrambled control tumors, especially with regard to vessel-associated VEGF (Fig 4G and 4H). It has been previously been shown that loss of VEGF results in poor tumor vascularization leading to severe hypoxia and restricted growth of tumors. Notably, we observed that tumors from scrambled-treated animals displayed little hypoxia, whereas in Tks5 KD tumors extensive areas of hypoxia were detected (Fig 4I, 4J and 4N). Together, our results support a novel role for Tks5 in promoting the growth of an extensive and functional tumor vasculature.

### Continued Tks5 expression is required for tumor growth

**Fig 5:**
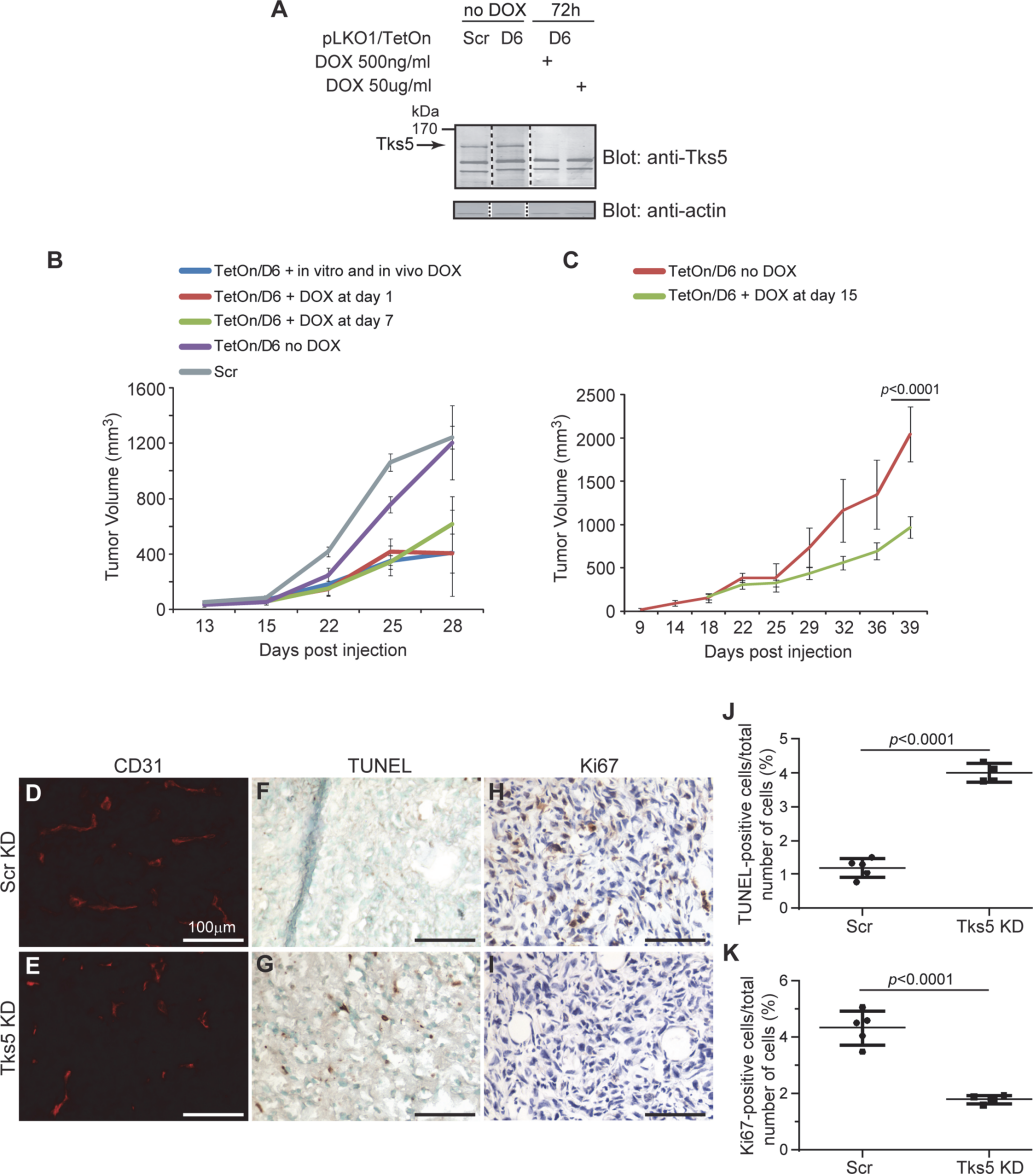
Tks5 is required for tumor progression. MDA-MB-231-Luc cells were infected to stably express an inducible TetOn lentivirus where the levels of the Tks5 shRNA are under the control of the tetracycline promoter (see Materials and methods for the experimental procedure). A) Immunoblot demonstrating Tks5 expression reduction in the MDA-MB-231-Luc cell line in dose- and time-dependent fashion in response to doxycycline exposure. B) TetOn/D6 were injected orthotopically (blue, red, and green lines) compared to SCR and no doxycycline controls (grey and purple lines, respectively) under three conditions: when Tks5 was already reduced by *in vitro* exposure of the cells to doxycycline for 10 days (DOX A) (blue line), when unexposed cells were injected into the animal and the animal received doxycycline starting at the day of injection (DOX B) (red line), as well as when the animals received doxycycline in the drinking water for the first time after the tumor has been growing for 7 days (DOX C) (green line). C) Animals were given doxycycline 15 days after tumor cell injection and after randomization of the mice. TetOn/D6 mice were divided up in 3 groups where 2 groups received doxycycline in the drinking water at different time points and 1 group received doxycycline-free drinking water. Tumor volumes were measured at different time points as described in Materials and methods, and tumors were allowed to grow to a final volume of approximately 2cm^3^. N = 4 mice per tumor type. Experiments were performed in duplicate. D-I) Tumors from Fig 4C were analyzed for vascularization by CD31 (panel D-E), for apoptosis by TUNEL (panel F-G) and for proliferation using Ki-67 (panel H-I) immunofluorescence staining. Scale bar: 100μm. Images are representative for all experiments performed. J-K) Quantification of positive immunohistochemical and immunofluorescence staining. Graphs show immunopositive cells for apoptosis (TUNEL) (panel J) and proliferative (Ki67) (panel K) markers at the day of dissection (endpoint of experiment). Data were expressed as mean ± SD. One-way ANOVA or a Student’s t test was used to calculate *p* values.

The poor tumor growth we observed in Tks5-KD cells could reflect either a requirement for Tks5 expression for the establishment of mammary tumors, or an ongoing requirement for Tks5 for tumor growth. To distinguish between these possibilities, we next generated a mouse mammary tumor model in which human Tks5 expression was doxycycline-regulated. MDA-MB-231-Luc breast cancer cells were infected to stably express an inducible TetOn lentivirus where the levels of the Tks5 D6 shRNA are under the control of the doxycycline promoter [18]. Prior to *in vivo* studies, we confirmed via immunoblotting that Tks5 knockdown in the TetOn/D6 cells occurred in a dose- and time-dependent fashion in response to doxycycline exposure (Fig 5A). Then we established three test groups. Group 1 consisted of TetOn/D6 cells exposed to doxycycline for 10 days *in vitro* prior to orthotopic injection into mammary fat pads, with continued exposure *in vivo* (Fig 5B). In Group 2 the TetOn/D6 cells were not exposed to doxycycline *in vitro*, but doxycycline was added to the drinking water starting on the day of orthotopic injection (Fig 5B). For Group 3, animals injected with TetOn/D6 cells were exposed to doxycycline in the drinking water starting at day 7 post-injection, once tumors were established (Fig 5B). Knockdown of Tks5 impaired tumor growth to a similar extent under all 3 conditions, compared to the growth of TetOn/D6 cells which were never treated with doxycyline (p<0.05 by two-way repeated ANOVA with Bonferroni post-hoc test). (Fig 5B). In addition, the tumors in all three treated groups were significantly smaller than the tumors formed by TetOn/D6 cells which were never treated with doxycycline (p<0.01 by two-way repeated ANOVA with Bonferroni post-hoc test). Similar results were also obtained when doxycycline treatment was delayed until 15 days after injection (Fig 5C). We further analyzed the tumors from this experiment histologically. We observed decreased vessel density in doxycycline-regulated TetOn/D6 tumors compared to untreated TetOn/D6 cells tumors (Fig 5D and 5E), as well as increased apoptosis (Fig 5F-5G and 5J) and decreased cell proliferation (Fig 5H-5I and 5K). To summarize, these studies reveal that continued Tks5 expression is required for tumor growth.

## Discussion

Our overall objective for this study was to test the hypothesis that Tks5 is a critical player in breast cancer tumor growth and progression *in vivo*. Significantly, a pioneering study on Tks5 in human cancer found a clinical prognostic relevance in glial-derived brain tumors [14] and prostate cancer [16], and a recent study defined the importance of a high ratio of Tks5α to Tks5β mRNAs in lung cancer [15]. Our own examination of Tks5 using tissue microarrays revealed an increased Tks5 expression in clinical breast cancer specimens, with approximately half of primary invasive tumors displaying high Tks5 protein expression. Analysis of RNA sequencing data did not reveal any correlation between total Tks5 mRNA, or high Tks5α:β ratios, and outcome. However, there was a significant association between expression of the long form, Tks5α, and survival, particularly for those diagnosed with stage I/II disease. Interestingly, this association was most pronounced in the population with late (> 5 year) recurrence of disease. This intriguing finding suggests a role for Tks5α beyond initial metastatic spread, and raises the possibility that Tks5, and/or invadopodia, might be involved in tumor dormancy. This could be consistent with the finding of reduced tumor angiogenesis, since some studies have linked the angiogenic switch to escape from tumor dormancy [26]. As a caveat, we note that the survival data in the TCGA dataset does not list cause of death, and it is highly likely that some patients will have died of causes other than breast cancer. Nevertheless, further interrogation of the clinical relevance of Tks5α in breast cancer is clearly warranted. To this end, we are currently preparing an antibody specific for the PX domain of Tks5 for use in immunohistochemical analyses.

We used RNA interference to test the role of Tks5 in tumor growth. While the shRNA sequences we used would target both the long (α) and short (β) forms of Tks5, we note that human cancer cells in general, and MDA-MB-231 in particular, express predominantly Tks5α ([8] and Fig 2). We found a marked inhibition of tumor growth in these experiments, regardless of shRNA sequence used, or whether knockdown was accomplished *in vitro* or after tumor establishment *in vivo*. These results are consistent with our previous findings using Src-3T3 cells [13], and melanoma cell lines (unpublished). However, they differ from published data on Twist-expressing human mammary epithelial cells [27] and mouse lung adenocarcinoma cell lines injected subcutaneously, where reduced metastasis, but no deficits in tumor growth at the injection site, were noted [15]. There are many possible explanations for these discrepancies. One possibility is that site of injection is important, since the data shown here were obtained with orthotopic injections, although our Src-transformed fibroblast experiments also used the subcutaneous route. Tumor and/or cell type differences may also provide an underlying reason. In addition, we measured tumor growth during the first weeks after implantation, whereas Eckert et al [27] and Li et al [15] only reported tumor weights at the end point of the experiment. Perhaps tumors eventually overcome the requirement for Tks5, or shRNA control of Tks5 expression is lost. One way to complement such knockdown xenotransplantation experiments and distinguish between these possibilities is to determine the role of Tks5 in a mouse model of breast cancer, which is currently underway. Regardless, because of the poor growth of the primary tumor we did not measure metastatic dissemination of the Tks5 knockdown MDA-MB-231 cells. However, our preliminary data suggest that when tumor cells were introduced directly into the circulation to circumvent the need for intravasation, metastatic growth in the lung was inhibited by Tks5 knockdown, which is in keeping with our previously published findings with Src-3T3 cells [13].

Why do tumors fail to grow when they lack Tks5? We noted less proliferation and more apoptosis, as well as increased hypoxia, within the tumors. This could perhaps all be explained by the aberrant angiogenesis we noted, characterized by thinner and leakier vessels. It is well established that the primary stimulus for angiogenesis in tumors is VEGF [28]. By immunohistochemistry, we detected a marked reduction in vessel-associated VEGF staining in the Tks5 knockdown tumors, which is consistent with the angiogenesis defects we observe, and also consistent with the concept of an angiogenic switch regulating tumor progression [29]. However, we have not noticed a reduction of VEGF expression in response to Tks5 knockdown *in vitro* (not shown). Future experiments will seek to address the reason for the reduced angiogenesis *in vivo*, by examining the tumorigenic properties of breast cancer cells expressing forms of Tks5 mutated to affect its binding to various effector proteins.

Are the effects we observed on tumor growth due to loss of invadopodia, or because of some other function of Tks5, independent of its activity as an invadopodia scaffold? At this point we suspect the former. For example, it is the long (α) form of Tks5 which is required for invadopodia formation [9,11,15], and the expression of this form correlates with poorer disease outcome. Furthermore, while the literature equates the presence of invadopodia with the first steps of the metastatic cascade, in particular intravasation, loss of other invadopodia proteins such as cortactin [30] and MT1-MMP [31] also has an effect on tumor progression. While cortactin and MT1-MMP are not found exclusively at invadopodia, complicating interpretation of these experiments, these data also support the possibility that loss of invadopodia function is the common feature of the tumorigenesis experiments. This might in turn suggest that the control of pericellular proteolysis, a key function of invadopodia, is at the heart of the phenotypes we observe. In keeping with this, VEGF, and other cytokines are growth factors, are processed to their mature and active forms by extracellular proteases. We are currently investigating this possibility in both the 3D and tumor growth assays described here.

## Conclusions

We find that the high expression of Tks5α, particularly in patients with stage I/II disease, is associated with a worse outcome, regardless of tumor subtype, particularly for those patients with late recurrence. RNA interference experiments showed that loss of Tks5 was associated with reduced tumor cell growth, both *in vitro* in 3D culture, and *in vivo* in orthotopic sites. Tks5 knockdown resulted in defective tumor angiogenesis, accompanied by reduced proliferation and increased apoptosis and necrosis of tumor cells. Taken together with reports from the literature on other invadopodia proteins, these data suggest that invadopodia play broader roles during tumor progression beyond intravasation and extravasation, to impact tumor growth. We speculate that the control of pericellular proteolysis, and subsequent effects on both protein processing and extracellular matrix degradation, might be the underlying mechanism. Data Availability: The authors confirm that all data underlying the findings are fully available without restriction. All relevant data are within the paper.

## Supporting Information

S1 ARRIVE Checklist NC3Rs ARRIVE Guidelines Checklist_Blouw et al. (PDF)
